# Crystallisation Dynamics in Large-Scale Extrusion Additive Manufacturing: An Analysis with and without Temperature Modification

**DOI:** 10.3390/ma17102243

**Published:** 2024-05-10

**Authors:** Dominik Leubecher, Steffen Brier, Pablo Vitale, Bruno Musil, Philipp Höfer

**Affiliations:** 1 Institute of Lightweight Engineering, University of the Bundeswehr Munich, 85577 Neubiberg, Germany; pablo.vitale@unibw.de (P.V.); bruno.musil@unibw.de (B.M.); philipp.hoefer@unibw.de (P.H.); 2 Institute for Machine Tools and Production Process, Chemnitz University of Technology, 09126 Chemnitz, Germany; steffen.brier@mb.tu-chemnitz.de

**Keywords:** large-scale additive manufacturing (LSAM), material extrusion (MEX), temperature field modification, macro- and microscale processes, crystallisation, simulation

## Abstract

Large-Scale Material Extrusion (LS-MEX) is increasingly being used in small-scale production and prototyping due to its ability to create components in new temporal and spatial dimensions. However, the use of this manufacturing process poses microscopic and macroscopic challenges not encountered in previous small-scale production systems. These challenges arise primarily from the prolonged retention of heat in the material, which leads to insufficient strength in the extruded strands at the macrostructural level. As a result, the component can collapse, a phenomenon known as ‘slumping’. Thermal energy also influences microstructural changes, such as crystallisation kinetics, which affect properties such as the strength and stiffness of the final product. The duration and dynamics of thermal energy are influenced by manufacturing parameters and the possible use of additional peripheral equipment, which affects component quality. In this study, the influence of thermal energy on structural processes through simulations of polyamide 6 with 40% carbon fibres (PA6 wt.%40 CF) is investigated. The results show that by adjusting the process parameters and using modification units, the thermal profile of the material can be accurately controlled, which allows the microstructural processes to be precisely controlled. This leads to the targeted modification of the macroscopic material properties. The focus of this work is on the combination of numerical simulations of the LS-MEX process with semi-empirical methods for the analysis of crystallisation processes. The application of the Nakamura model, which is used throughout similar investigations, allows a detailed description and prediction of the crystallisation kinetics during the manufacturing process. The study shows that the absolute degree of crystallisation can be determined with simplified assumptions using a combination of thermal simulations and semi-empirical approaches. It was found that the absolute degree of crystallisation increases from the outer interface of the strand to the print bed across the cross-section. This can be attributed to the specific thermal boundary conditions and the resulting temperature profiles at different points.

## 1. Introduction

The use of Large-Scale Material Extrusion (LS-MEX) systems in small series production and prototyping has increased due to the ability to produce complex components in new temporal and spatial dimensions [1,2]. However, compared to conventional small-scale manufacturing approaches, the LS-MEX process presents challenges at both the microscopic and macroscopic levels. At the macroscopic level, the challenges are considered to be directly related to the overall structure and shape of the material or component, thus affecting its functionality. For example, the prolonged storage of thermal energy in the material can lead to insufficient strength of the extrusion strands, which in some cases results in the collapse of the component, a failure mode known as ‘slumping’ [3,4]. At the microscopic level, prolonged thermal energy affects processes at the molecular level, such as crystallisation. This in turn significantly affects the mechanical properties of the final product. These microscale processes are critical in determining the strength, stiffness and other fundamental mechanical properties of the material [5,6].

A critical factor in the challenges of LS-MEX is the dynamics of the thermal energy in the material, which is significantly influenced by process parameters such as layer height and deposition speed. LS-MEX allows the use of layer widths and heights that are more than ten times larger than those used in small-scale production systems. This scaling results in cooling times ranging from seconds to several minutes, making thermal management very different from that of the small-scale process [7]. The increase in deposited volume, combined with the low thermal conductivity of thermoplastics, means that the heat energy supplied remains stored in the material for a longer time. In addition, the larger volume requires the consideration of the temperature gradient in the material, which can be quantified by the Biot number [8,9,10].

The energy input into the manufacturing process is determined by the process parameters and can be modified by the use of additional peripherals. Various peripherals for heating, such as lasers or radiant heaters, and for cooling, such as fans, have been successfully used in small- and large-scale manufacturing processes [11,12,13,14,15]. Specifically, in the context of the LS-MEX process, Tagscherer et al. [15] observed that the targeted use of such auxiliary devices, in particular an infrared heater as a heating element and compressed air as a cooling element, can contribute to an improvement in the mechanical properties of the components during the process. This improvement is thought to be due to changes at the microstructural level, for example, in crystallisation kinetics. It is known that molecular diffusion is more efficient in amorphous regions than in crystalline regions; the resulting diffusion of molecular chains and the associated intermolecular interaction forces lead to an increase in the strength of the final product [16]. It is, therefore, essential to characterise the crystallisation kinetics in detail and to investigate the relationship between these microstructural features and the resulting properties of the final product. The knowledge gained from this investigation will allow the crystallisation processes to be specifically influenced by careful selection of the manufacturing process parameters or by the use of additional peripheral devices such as heating and cooling elements.

Various models in the literature [17,18,19] describe the crystallisation kinetics of semi-crystalline thermoplastics based on experimental data from differential scanning calorimetry (DSC). Dörr et al. used a modified Nakamura model to describe the non-isothermal crystallisation kinetics, as described in detail in [17]. To calculate the crystallisation kinetics, the associated differential equation is solved using the backward Euler method. The parameters required for the equation, such as the Avrami constant, were determined using a global optimisation method and adjusted for different cooling rates. The study shows that the description of crystallisation is strongly dependent on the cooling rates, which underlines the importance of the temperature fields occurring during the process. Kulkarni et al. [6] used a similar approach to describe the crystallisation kinetics but determined the required parameters using a local optimisation method. In addition, they integrated information about the crystallisation kinetics and the thermal energy released in the process into their thermal simulation model to provide a more holistic view.

In this paper, a combination of numerical modelling of the LS-MEX process using the finite element method (FEM) and semi-empirical approaches to crystallisation kinetics based on the Nakamura model allows the microstructural processes in the manufacturing process to be accurately determined. Historical data on crystallisation kinetics allow correlations to be established for predicting the mechanical properties of the final product without having to rely on extensive experimental test series. The exact correlation is not presented here but is the subject of further research.

The thermal simulation of the manufacturing process takes into account the real boundary conditions of the process [7,20,21] and also offers the possibility of integrating additional peripheral devices such as heating and cooling elements. The main objective is to study the dynamics of crystallisation taking into account different production parameters or the use of peripheral equipment. In particular, the influence of heating and cooling elements on the thermal profiles within an extrudate strand is analysed, with specific crystallisation processes being considered in detail.

A central research focus of the current study is to investigate the influence of different thermal conditions on crystal morphology and velocity. These factors limit molecular diffusion at the interface of the extrudate strand and can, therefore, influence the mechanical properties of the final product. The influence of these variables on coating adhesion is not discussed further in this manuscript but remains a target for future research. It should be noted that experimental investigations to confirm the theoretically determined crystallisation curves are not presented in this work. The focus is primarily on the numerical determination of crystallisation kinetics in LS-MEX processes. In addition, the heat development induced by crystallisation as a heat sink in the material is not included in the simulation in the current work. The material investigated in this study is polyamide 6 reinforced with 40%-by-weight carbon fibres (PA6 wt.%40 CF—AKRO Compounds).

## 2. Methods

### 2.1. Thermal Simulation of LS-MEX

The FEM-based thermal simulation of the LS-MEX process is characterised by the time- and location-dependent activation and deactivation of elements using the “element-birth-and-death” technique [22] and takes into account the thermal boundary conditions [6,14,15]. In addition to the thermal energy introduced by the temperature-controlled screw extruder during extrusion, the three heat transfer mechanisms—conduction, convection and radiation—are crucial for understanding the thermodynamic processes in the material. The partial differential equation of heat conduction and the specific boundary conditions of the process are formulated in detail for the analytical description of the MEX process as follows:

The temperature field $T\left(\vec{x},t\right)$ is defined by the heat conduction equation

(1) $$\rho c_{p}\frac{\partial T(\vec{x},t)}{\partial t}=\nabla \cdot [\lambda_{0}\nabla T(\vec{x},t)]$$

where ρ [$kg/m^{3}$] is the material density, $c_{p}$ [J/(kgK)] is the specific heat capacity and $\lambda_{0}$ [W/(mK)] is the thermal conductivity of the material used. The temperature is adjusted to the extrudate temperature $T_{Ext}$ [K] according to the time-dependent activation of new elements at defined positions, which serves as a Dirichlet boundary condition for the added elements Ω of the strand.

(2) $$T\left(\vec{x},t\right)=T_{Ext};\vec{x}\in \Omega$$

Heat conduction occurs when there is a temperature gradient, ∇T [K/m], between deposited materials or between the deposited material and the defined temperature of the print bed at the corresponding surface, represented by $\Omega_{Cond}$. Fourier’s law of heat conduction can be formulated as

(3) $${\vec{q}}_{cond}=-\lambda_{0}\cdot \nabla T\left(\vec{x},t\right);\vec{x}\in \Omega_{Cond}$$

where ${\vec{q}}_{cond}$ [$W/m^{2}]$ is the heat flux density, which describes the direction and amount of heat energy flowing through the material per unit time and per unit area.

Convective and radiative heat transfer mechanisms influence the temperature field of the exposed extrudate and print bed surfaces, $\Omega_{Conv+Rad}$, and are applied using Neumann boundary conditions. The integration of radiative heat transfer into commercial FEM software, such as ANSYS Workbench 2021 R2, is challenging when using the ‘element-birth-and-death’ technique and requires a modification of the convective heat transfer coefficient to include the effects of radiative heat transfer [23,24]. The combined heat transfer coefficient $h_{Tot}$ [$W/(m^{2}K)]$ is composed of the convective heat transfer coefficient $h_{Conv}$ [$W/(m^{2}K)]$, based on Newton’s law of cooling, and the radiative heat transfer coefficient $h_{Rad}$ [$W/(m^{2}K)]$, derived from the Stefan–Boltzmann law [24]. The calculation of $h_{Tot}$ is carried out according to the corresponding equation:

(4) $$h_{Tot}=h_{Conv}+h_{Rad}$$

The following equation is used to calculate the heat loss in the thermal system due to convection and radiation:

(5) $$\begin{array}{l} \;\;\;\;\;\;\;\;\;\;\;-\lambda_{0}\cdot \nabla T\left(\vec{x},t\right)=h_{Tot}\cdot \left(T_{Surf}-T_{Amb}\right)\\ \;\;\;\;\;\;\;\;\;\;\;\;\;\;\;\;\;\;\;\;\;\;\;\;\;\;\;\;\;\;\;\;\;\;\;\;\;\;\;\;=h_{Conv}\cdot \left(T_{Surf}-T_{Amb}\right)+\varepsilon \cdot \sigma \cdot \left[T_{Surf}^{4}-T_{Amb}^{4}\right];\;\vec{x}\in \Omega_{Conv+Rad} \end{array}$$

The combined heat transfer coefficient $h_{Tot}$ can be formulated as follows:

(6) $$h_{Tot}=h_{Conv}+\varepsilon \cdot \sigma \cdot \left\{\left[T_{Surf}+T_{Amb}\right]\cdot \left[T_{Surf}^{2}+T_{Amb}^{2}\right]\right\}$$

The convective heat transfer coefficient $h_{Conv}$ is determined by making certain assumptions about the type of convection and geometric characteristics. In this context, natural convection on a cylindrical body is assumed, on the basis of which, the heat transfer coefficient is determined according to the dimensions of the extruded strand [25]. The indirect heat transfer coefficient $h_{Rad}$ considered for the radiation results from the emissivity ε [−] and the Stefan–Boltzmann constant σ [$W/{(m}^{2}K^{4})]$. This coefficient also depends on the ambient temperature $T_{Amb}$ [K] and the temperature $T_{Surf}$ [K] at a given location and time on the free surface of the material.

**Figure 1:**
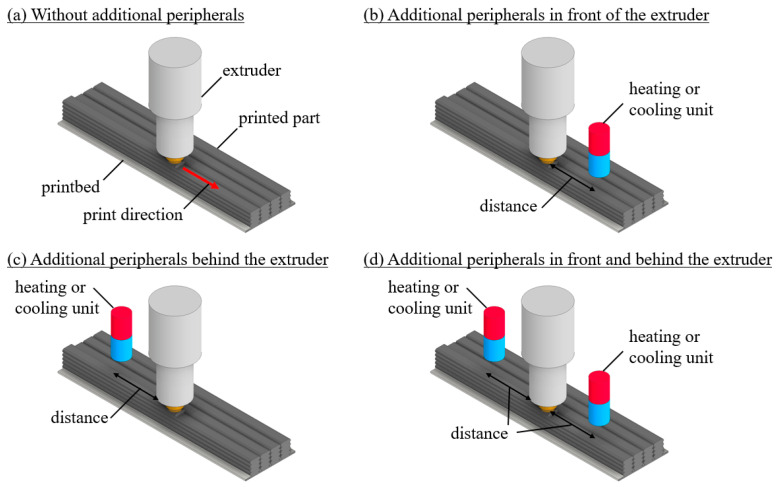
LS-MEX process with different configurations.

Additional peripherals, including heating and cooling elements, are integrated into the thermal simulation model to influence the thermal field during the process. Four different configurations are implemented: (a) process representation without additional peripherals, (b) process representation with additional peripherals in front of the extruder, (c) process representation with additional peripherals behind the extruder, and (d) process representation with additional peripherals both in front of and behind the extruder, see Figure 1. The heating and cooling elements act on the heat field by convection. Specifically, a fan heater is used as the heating element, which heats the air by convective heat transfer and by means of a fan, thereby changing the temperature field of the extrudate strand in a targeted manner. The cooling element also changes the temperature field by generating a cool air stream, resulting in accelerated cooling of the strand. These configurations allow flexible positioning of additional peripheral devices that only affect the surface of the extrudate strand facing the device. Of particular interest is the surface of the extrudate strand on which the next strand is placed, either next to or on top of it. These peripheral devices act on a rectangular area of 13.5 mm × 13.5 mm. In the simulation, the distance between the peripheral devices and the extrudate strand can be varied and should take on realistic, practical values. Details of the configurations, intensities and distances of these devices from the die for the numerical analysis are given in Table 1.

From a cost perspective, all variations can be covered equally. The experimental test fixture must therefore be designed so that the peripherals can be used both in front of and behind the nozzle. The variation does not lead to different costs. The quality of the implementation depends on the path guidance, the coverage by the peripheral units, the design and specification of the units and the design of the adapter.

The numerical integration of the heating and cooling elements is achieved by applying defined heat flux densities ${\vec{q}}_{HP}$ [$W/m^{2}]$ for heating elements and ${\vec{q}}_{CP}$ [$W/m^{2}]$ for cooling elements on the substrate surface $\Omega_{Sub}$ of the extrudate strand. Since these peripheral devices introduce heat energy into the system by convection, the process can be formalised as follows

(7) $$\begin{array}{l} {\vec{q}}_{HP/CP}=-h_{Conv}\cdot \left(T_{Surf}-T_{Amb}\right)\cdot \vec{n,}\\ \mathrm{for}\;\mathrm{heating}\;\mathrm{element}\;(HP)\;\mathrm{and}\;\mathrm{cooling}\;\mathrm{element}\;(CP) \end{array}$$

where $\vec{n}$ [−] is the unit normal vector at the surface indicating the direction of the heat flow. The negative sign in Equation (7) takes into account that the heat flux densities are convectively introduced into the thermal system, which corresponds to an input or extraction of thermal energy.

**Figure 2:**
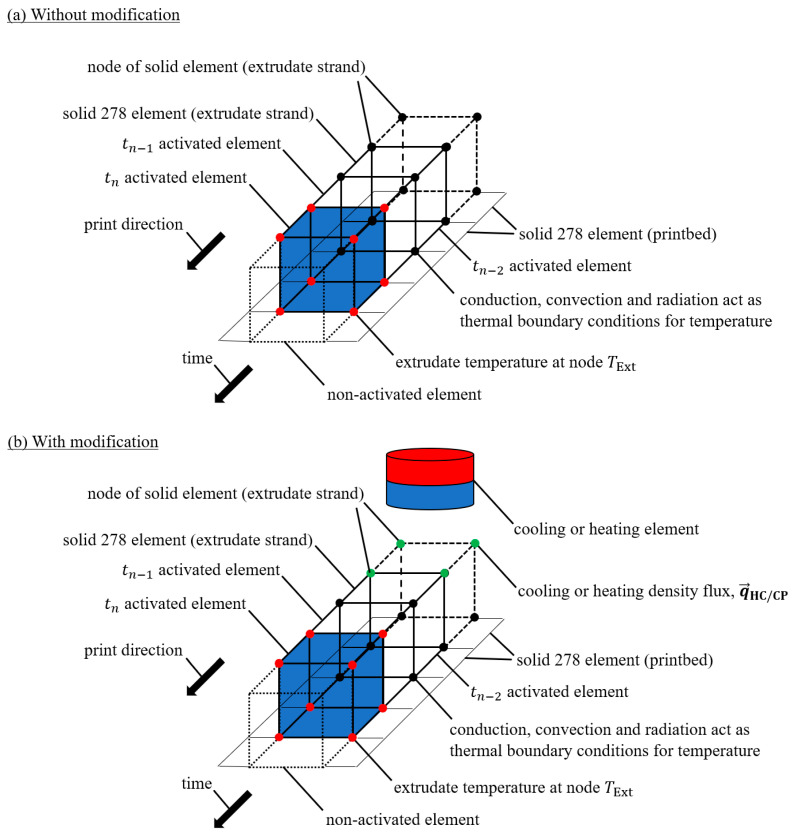
Conceptual illustration of the application of thermal constraints in the printing process: (**a**) without modification devices and (**b**) with modification devices. The technique of ‘element-birth-and-death’ is also illustrated. The red nodes represent the applied extrudate temperatures $T_{Ext}$. The black nodes represent the nodes where decreasing temperatures prevail over time and where the temperature boundary conditions—conduction, convection and radiation—are effective. The green nodes illustrate the overriding of the thermal boundary conditions by the modification units.

In the simulation, the boundary conditions at selected nodes are modified by the predefined heat flux densities of the heating and cooling elements, overriding the primary convection and radiation conditions. These adjustments vary according to the implemented configuration and are made at fixed distances from the extruder. The specific applications of these thermal constraints are shown in Figure 2.

### 2.2. Material Polyamide 6 Reinforced with 40 wt.% Carbon Fibres

This study investigates the crystallisation kinetics of polyamide 6 reinforced with 40 wt.% carbon fibers (PA6 wt.%40 CF), which was purchased from AKRO Compounds. This semi-crystalline engineering thermoplastic is characterised by high mechanical strength and stiffness, making it suitable for a wide range of industrial applications [26].

In the LS-MEX process, the addition of carbon fibres not only improves the mechanical properties but also the processability [27]. The reduction in residual thermal stresses due to the use of carbon fibres enables the production of large components, as the fibres absorb the stresses generated during the process. The fibres are mainly oriented in the direction of printing but also show swirls.

However, the carbon fibres in the material act as nucleating agents, influencing both the rate and morphology of crystallisation [28]. This can be particularly detrimental at interfaces, where the crystallisation process is promoted, crystalline structures are formed and the diffusion of molecular chains can be restricted.

### 2.3. Procedure for the Experimental Analysis of Crystallisation Kinetics

To investigate and characterise the crystallisation kinetics of the employed material, experimental analyses were carried out using a power-differentiated DSC instrument in accordance with the guidelines of DIN EN ISO 11357-7 [29]. The DSC instrument, a Perkin Elmer DSC 8500, allows cooling and heating rates of up to 750 K/min and operates with a nitrogen flow of 20 mL/min as an inert gas. Samples are analysed in a perforated aluminium cuvette supplied by the manufacturer of the DSC. Due to the addition of carbon fibres, the sample masses are selected so that a mass of 6 mg of the thermoplastic material can be analysed. Due to the variable cooling and heating rates in the non-isothermal LS-MEX process, experiments on non-isothermal crystallisation are carried out.

**Figure 3:**
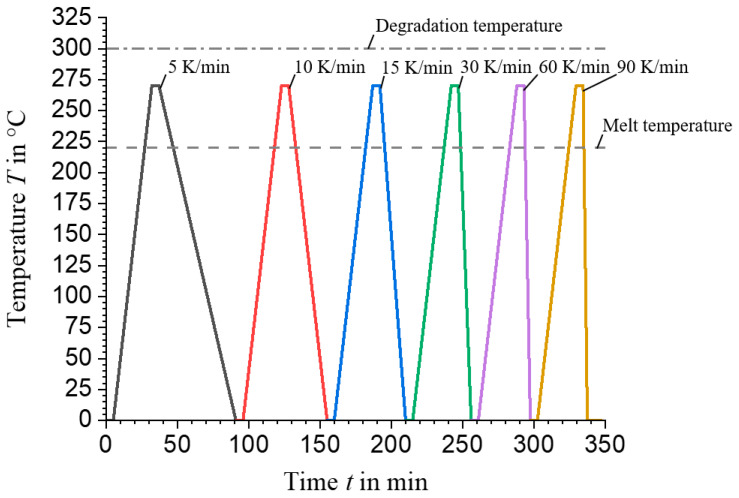
Method for determining the non-isothermal crystallisation of PA6 wt.%40 CF: The heating rate is a constant 10 K/min and the exothermic heat flows have been determined for the cooling rates shown. The melting temperature of the material is around 220 °C, while degradation begins above 300 °C. This shows that the calorimetric tests were carried out within an acceptable temperature range. To ensure the thermal stability of the sample between the heating and cooling phases, isothermal hold times of 5 min were introduced.

Constant heating rates of 10 K/min and variable cooling rates (5, 10, 15, 30, 60, up to a maximum of 90 K/min) are applied, as shown in the test programme in Figure 3. Five different samples are statistically analysed. The crystallisation curves are accurately recorded using experimentally determined heat flow curves and a specially developed script in Matlab R2021a to determine crystallisation kinetics.

The procedure begins with the extraction of the crystallisation peak from the heat flow curve and the determination of the associated linear baseline using the script. This is followed by the integral determination of the enthalpy of the crystallisation peak ($H_{\infty }\;$[J/g]) and the stepwise division by the partial areas of the peak ($H_{\Delta t}$ [J/g]), resulting in the relative crystallisation curves $X_{rel,exp}$ [%] [6,30].

(8) $${Χ}_{rel,exp}=\frac{H_{\Delta t}}{H_{\infty }}\cdot 100\%$$

To calculate the absolute crystallisation curves ($X_{abs,exp}$ [%]), the incremental partial area is divided by the enthalpy of the hypothetical, 100% crystallised material ($H_{100\%}\;$[J/g]). According to literature data [31], this value is 188 J/g for pure PA6.

(9) $${Χ}_{abs,exp}=\frac{H_{\infty }}{H_{100\%}}\cdot 100\%$$

### 2.4. Semi-Empirical Characterisation of Crystallisation Kinetics

In the present study, the crystallisation kinetics of the investigated material is analysed using the well-established Nakamura model [6,17,18,19]. This model allows the description of non-isothermal crystallisation in its differential form:

(10) $$\frac{dΧ}{dt}=n\cdot K(T)\cdot (1-Χ)\cdot {\left[-ln(1-Χ)\right]}^{1-\frac{1}{n}}$$

where X [%] is the degree of crystallisation, n [−] is the Avrami constant and K(T) [1/s] is the crystallisation constant as a function of temperature.

According to [32], which shows that crystallisation takes place in two steps—primary and secondary crystallisation—the differential form of the Nakamura model is applied twice (m=2) in the calculation:

(11a) $$Χ=\frac{1}{m}\sum_{m}{Χ}_{m};m\in N$$

(11b) $$\frac{d{Χ}_{m}}{dt}=n_{m}\cdot K_{m}(T)\cdot (1-{Χ}_{m})\cdot {\left[-ln(1-{Χ}_{m})\right]}^{1-\frac{1}{n_{m}}}$$

The aim of this study is to accurately determine the unknown parameters of the temperature dependence of the crystallisation constant K(T) and the Avrami constant n. For this purpose, advanced non-linear optimisation methods are used, in particular the Newton–Gauss algorithm and its variant, the ‘Trust-Region-Reflective’ algorithm. This choice is based on the robustness, flexibility and efficiency of the Trust-Region-Reflective algorithm in solving optimisation problems with linear constraints and non-linear models. The application of these methods permits to fit the simulation curves to experimentally determined data by minimising the sum of the quadratic deviations.

Compared to other approaches described in the literature, this study uses the same model to describe the kinetics according to Nakamura. However, in this manuscript, the method is simplified by assuming that n and K(T) are constant. In addition, two evolution equations are used to account for both primary and secondary crystallisation.

After adjusting the constant parameter n and the temperature-dependent material function K(T), it is possible to describe crystallisation curves for different cooling rates. The integration of these curves with the thermal data from the process simulation allows the prediction of the crystallisation kinetics at different points in the process. This integration highlights the ability to predict material behaviour under different conditions and to improve the understanding of crystallisation processes in temperature-sensitive materials.

### 2.5. Simulation Model Setup

To enable the simulation of the LS-MEX process, it is necessary to implement the boundary conditions that occur in reality. The ANSYS Workbench 2021 R2 software is used for this purpose, supplemented by the use of ANSYS Parametric Design Language (APDL) snippets. The simulation integrates the thermal boundary conditions according to Section 2.1, using SOLID278 elements. These elements are capable of modelling all heat transfer mechanisms.

The required values for the thermal boundary conditions are partly based on the selected experimental example, including the extrudate temperature $T_{Ext}$ [K]. The values for convection and radiation are calculated according to Section 2.1 or determined experimentally. The intensities of the heating and cooling elements are based on data available in the literature and are adapted to the specific requirements of this study, as shown in Table 1. The distance between the nozzle and the periphery $s_{NP}$ [m] is determined independently.

To ensure high accuracy with optimal computational time, the elements ($M_{L},\;M_{W}\;\mathrm{and}\;M_{H}\;[m]$) and time steps (∆t [s]) are carefully discretised, the specific values of which are documented in Table 1. A detailed illustration of the element sizing is also provided in Figure 5. This methodological approach confirms its suitability due to the high congruence achieved between experimental and numerical results, as explained in Section 3.1.

**Figure 4:**
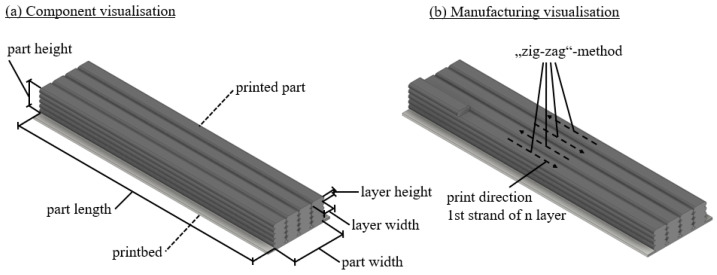
Schematic representation of (**a**) the geometry dimensions and (**b**) the production process of the exemplary model.

The example part is produced using the ‘zig-zag’ process as shown in Figure 4. This figure also shows the layer widths ($L_{W}$ [m]) and heights ($L_{H}\;[m]$) as well as the geometric dimensions of the part. The material used for the part is PA6 wt.%40 CF, while the material used for the pressure bed is wood. This choice of material is based on the improved adhesion due to the rougher surface of the wood in real tests.

**Figure A1:**
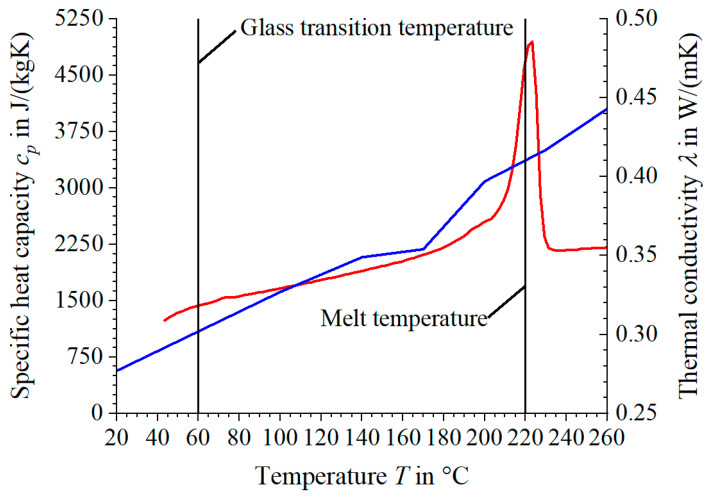
Curve of the specific heat capacity (red) and thermal conductivity (blue) for the material PA6 wt%40 CF. Specific heat capacity by experimental measurements using a DSC system Netzsch DSC 204 F1 Phoenix and thermal conductivity data provided by the company Akro-Compounds.

The thermal properties of the wood (thermal conductivity and specific heat capacity) are taken from the ANSYS material library, while those of the PA6 wt.%40 CF material are determined experimentally, see Table A1 and Figure A1.

## 3. Results

### 3.1. Verification of the Thermal Simulation Results

**Figure 5:**
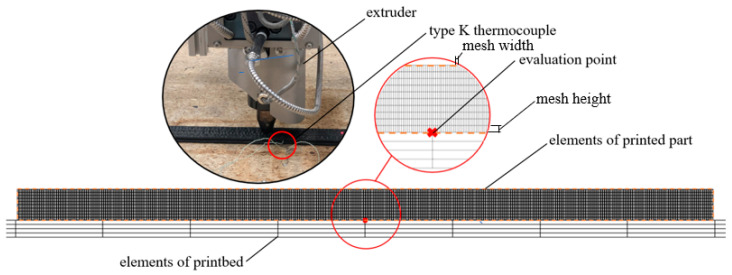
Representation of the evaluation point for thermal process simulation validation.

To verify the accuracy of the thermal process simulation, an experimental validation is carried out using the same production parameters as in Table 1. A calibrated type K thermocouple, with a diameter of 0.5 mm, supplied by TC Mess- & Regelungstechnik GmbH, is placed in the position shown in Figure 5—in the center of the first extrusion strand, halfway along the component—and the temperatures are measured at a rate of 10 Hz.

The recorded data are analysed using CatmanAP Version 5.5, with the evaluation being based on the average values and standard deviations obtained from five measurement cycles.

**Figure 6:**
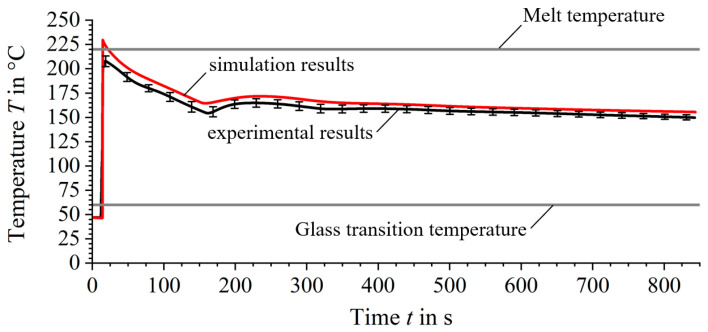
Thermal profile comparison at the evaluation point between experimental (black) and simulated (red) results.

When compared with the simulation results, the experimental results show remarkable agreement over the entire production period, see Figure 6. The initially lower experimentally measured temperatures of the deposited extrudate strand can be attributed to both the thermal inertia of the thermocouple [33] and the formation of cavities between the thermocouple and the extrudate strand.

In addition, the repeated temperature peaks that occur during the process are well modelled in the simulation results. The average deviation of the simulated curve from the experimental mean value is less than 5%, underlining the model’s suitability.

### 3.2. Crystallisation Kinetics of Polyamide 6 wt.%40 Carbon Fibres

**Figure 7:**
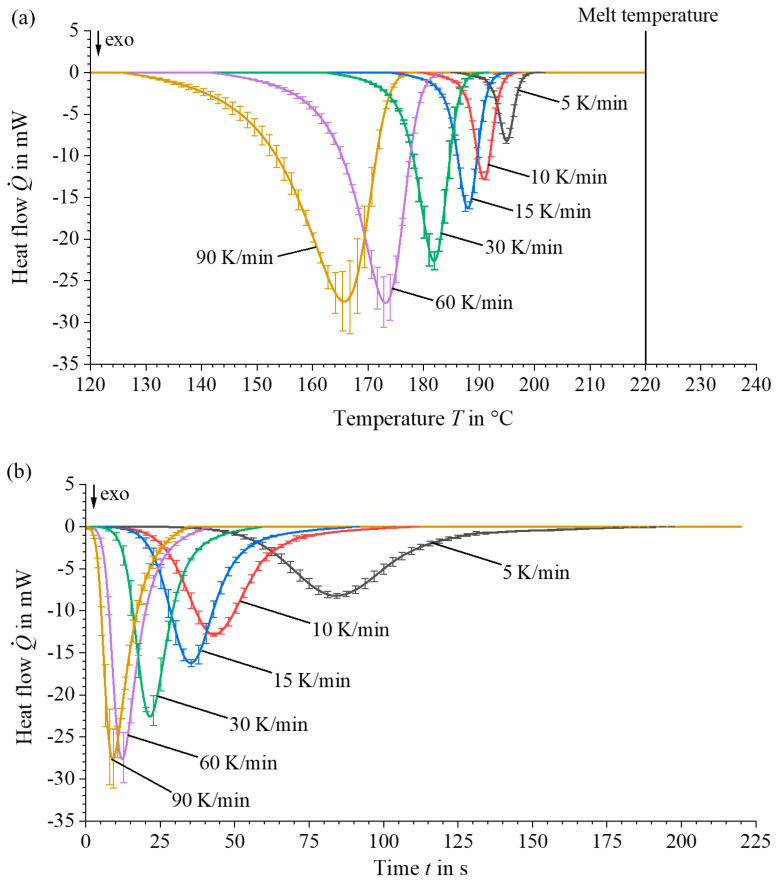
DSC analyses of the heat flow curves of PA6 wt% 40 CF in relation to (**a**) temperature and (**b**) time.

**Figure 8:**
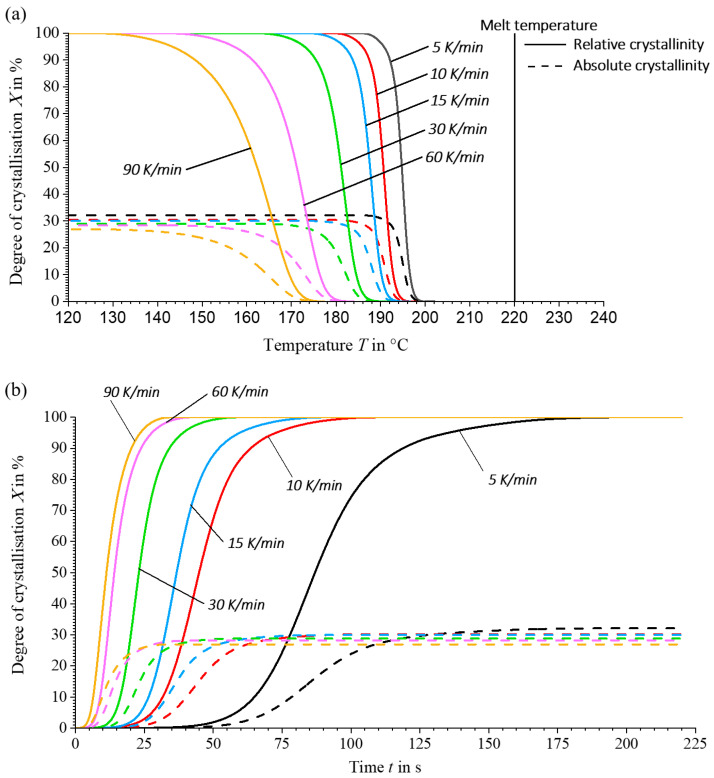
Relative (solid) and absolute (dashed) crystallisation process as a function of (**a**) temperature and (**b**) time.

In order to characterise the crystallisation kinetics of the material used, non-isothermal DSC tests are carried out at cooling rates of 5, 10, 15, 30, 60 and 90 K/min in accordance with Section 2.3. The analysis of the recorded curves shows consistent results with those reported in the literature [5,34]. As the cooling rate increases, the onset of crystallisation starts at lower temperatures and accelerates, as shown in Figure 7. At the same time, the enthalpy released during crystallisation decreases with increasing cooling rates, resulting in a lower absolute degree of crystallisation at higher cooling rates, see Figure 8 and Equation (11). These observations underline the significant effects on the crystallisation rate and the achievable degree of crystallisation, which are significantly influenced by the thermal gradients during the process.

The results in Figure 8 show that the material has sufficient time for complete crystallisation at lower cooling rates. Due to the rapid crystallisation tendency of PA6, there is a risk at very high cooling rates that the time for complete crystallisation is not sufficient, which could lead to cold crystallisation during reheating [35]. However, this effect is not observed at the cooling rates analysed. In the context of the LS-MEX process, in which very slow cooling rates prevail, this is not a critical limitation.

Modelling the crystallisation process for any temperature profile using a semi-empirical method is necessary to describe the crystallisation kinetics in the context of the whole transient thermal process, see Section 2.4. According to the described semi-empirical approach, the crystallisation kinetics for different temperature profiles are determined by parameter fitting and the backward Euler method. The Newton–Gauss algorithm with the ‘Trust-Region-Reflective’ method is used as an optimiser.

**Figure 9:**
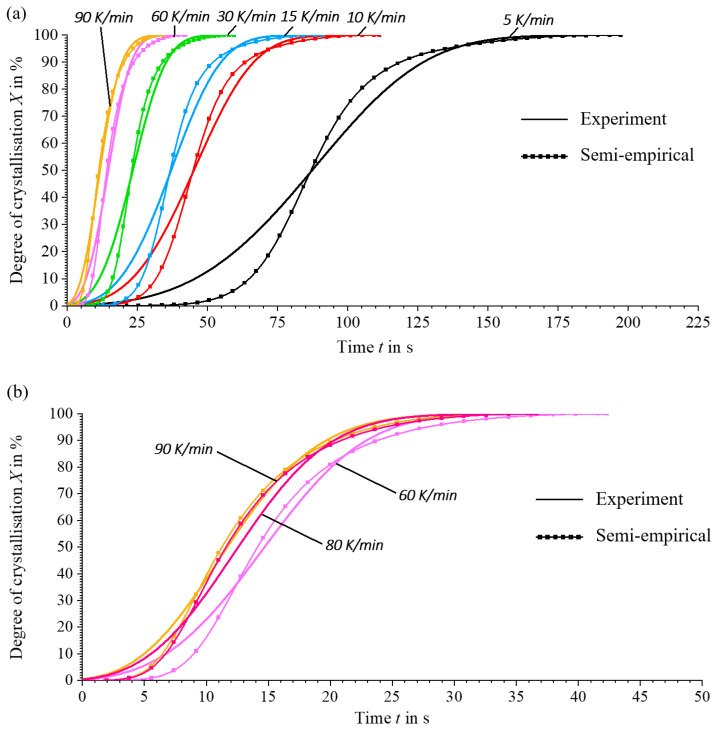
(**a**) Experimental and semi-empirically determined curves of relative crystallisation kinetics at varying cooling rates and (**b**) determination of the curves at unknown cooling rates.

A comparison of the experimentally observed crystallisation behaviour with the corresponding semi-empirical simulations in Figure 9 shows a deviation between the sigmoidal curves of the crystallisation processes at different cooling rates. This discrepancy results from the fact that the parameters determined for the parameter fit are assumed to be constants, which does not adequately reflect the actual curve behaviour. In particular, the temperature-dependent parameter K(T), which changes with temperature, is not integrated into the current method, leading to insufficient agreement with the experimental results.

Although [2] assumes a function similar to a Gaussian distribution for the course of the temperature-dependent crystallisation constant K(T), this does not reflect the progress due to an asymmetrical peak curve. Therefore, n and K(T) are initially assumed to be constant.

**Figure A2:**
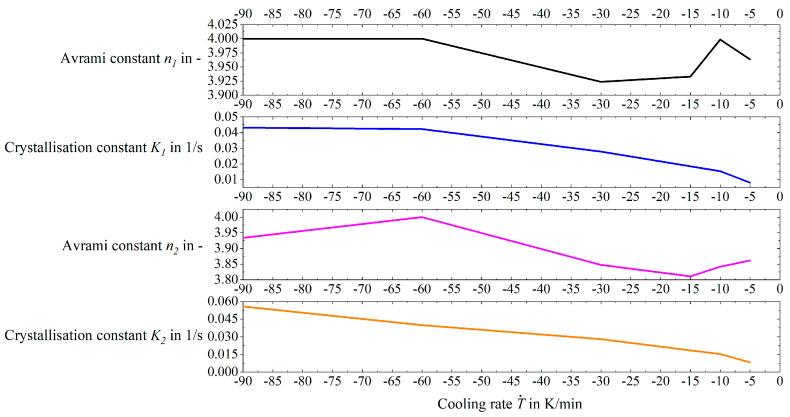
Parameters of the Nakamura bimodal crystallisation model: Avrami constants ($n_{1}$ and $n_{2}$) and crystallisation constants ($K_{1}\left(T\right)$ and $K_{2}\left(T\right)$).

Nevertheless, this method provides the first satisfactory results for making fundamental statements about crystallisation kinetics of all the literature reviewed. This method also allows the crystallisation processes to be determined for different thermal profiles and thus the crystallisation kinetics for previously unknown cooling rates as shown in Figure 9, e.g., 80 K/min. The results of the parameter fit for the cooling-rate-dependent parameters n and K(T) are shown in Figure A2 in the Appendix.

The values of the temperature-dependent crystallisation constant K(T) show a continuous increase with increasing cooling rates, whereas the Avrami constants n show discontinuous changes depending on cooling rates. There could be several reasons for this:

Firstly, due to the local optimisation approach, the determined parameters could only represent the local minima of the differential equation.

Secondly, the definition of the crystallisation constant as a fixed variable could influence the parameters of the Avrami constants in such a way that discontinuous jumps result. Theoretically, however, the progressions over the cooling rates should be continuous. This suggests that the assumption of a temperature-dependent constant K(T) as a fixed parameter is not precise enough to adequately describe the crystallisation processes. Nevertheless, this approach can be used for a preliminary description.

### 3.3. Modification of the Temperature Profiles through Additional Peripherals

The crystallisation dynamics and morphology within the microstructural processes are highly dependent on the selected production parameters and the use of ancillary equipment. Changes in the temperature profiles at specific points in the material result in different crystallisation rates and structures. In this section, the influence of such peripheral equipment on the temperature fields at different local points in the LS-MEX process is analysed. It is shown that heating and cooling elements can significantly modify the temperature profiles. Different configurations are analysed, which are explained in Section 2.1, and implemented according to the parameters in Table 1. For this purpose, the thermal profiles of three material points along the cross-section of an extrudate strand are analysed—at the validation site as described in Section 3.1. The thermal profiles at these points are compared: once for a component produced under standard production parameters without additional peripherals and once with these peripherals included according to Table 1.

**Figure 10:**
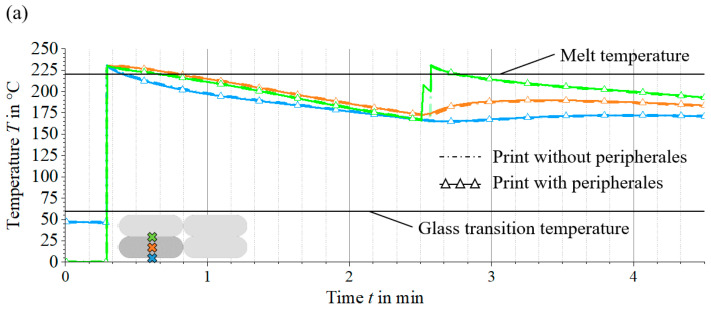

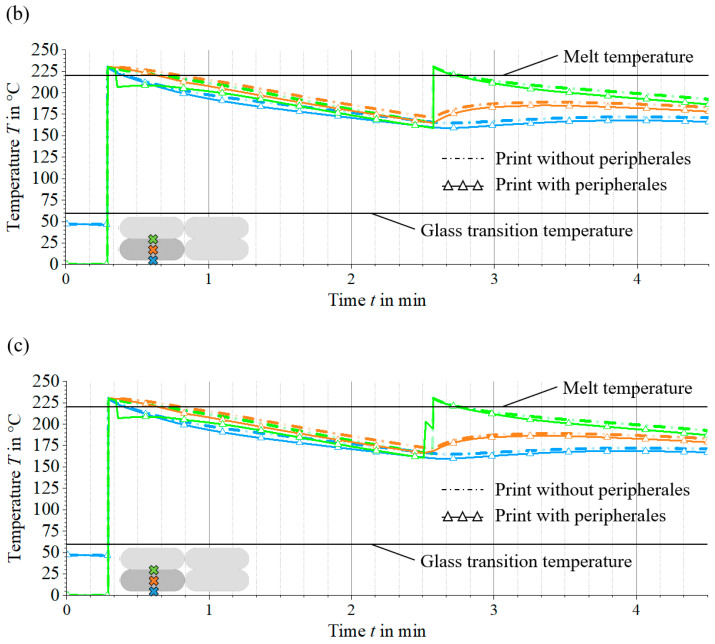
Thermal profiles for different material points in the extrudate strand: near the print bed (blue), strand center (orange) and on the substrate surface (green)—use without (solid line) and with additional peripherals (solid line with triangles superimposed). Variants: (**a**) additional periphery before the extruder (heating element), (**b**) additional periphery after the extruder (cooling element) and (**c**) use of two peripheries before (heating element) and after (cooling element) the extruder.

In the first modification, a heater with a power density defined in Table 1 is simulated in front of the extruder. The comparison of the thermal profiles at different points of the extrudate strand shows no relevant differences in the curves. The thermal profiles of the simple printing process are very similar to those of the modified process. There is only a slight difference in temperature over time as shown in Figure 10a. In addition, the previous element heats the material before a new extrudate strand is deposited. This additional heating can influence the crystallisation kinetics and increase the adhesion between two extrudate strands by melting or briefly vibrating crystalline structures that have already formed. The new strand of material deposited by the nozzle positioned after the heater causes a rapid rise in temperature at the evaluation point, followed by a subsequent drop in temperature. Experimental studies have shown that premature heating of the substrate layer improves the mechanical properties of the final product [12,15].

The second modification, with the periphery positioned behind the extruder, incorporates a cooling element as shown in Table 1. A comparison of the thermal profiles at different points of the extrudate strand shows a noticeable difference that develops over time. The use of the cooling element results in accelerated cooling, as evidenced by the faster temperature reduction compared to the standard production process. In addition, the presence of the cooling element on the extrudate surface allows rapid cooling, as shown in Figure 10b, followed by reheating by the internal thermal energy of the strand. This modification of the temperature field has a decisive influence on the crystallisation kinetics. A rapid drop in temperature can affect the formation of crystalline structures and lead to recrystallisation on reheating. Amorphous structures improve adhesion between extrusion strands and increase component quality through more effective diffusion of molecular chains, as demonstrated in [16,36,37].

The last modification adds peripheral elements both before and after the extruder. The first and second modifications integrate a heating and a cooling element with known power densities. A comparison of the thermal profiles of this type of modification with the already known effects of the peripheral elements (see Figure 10a,b) shows that the combination of both modifications leads to a synergy of effects. The impact of these effects on the microstructure has already been described for the two previous modifications.

The targeted use of additional peripheral devices modifies the temperature field at different locations in the material and thus enables the crystallisation kinetics to be controlled. This modification of the thermal processes influences the course of the crystallisation process. The influence of these changes on the crystallisation processes, in particular the modification of the thermal fields, is not considered further in this manuscript as a semi-empirical model of the crystallisation kinetics is required for both cooling and heating. This is not addressed in this manuscript and requires further research. Figure 10 illustrates only the influence of the temperature fields in the process and shows the resulting change in the course of the crystallisation process.

### 3.4. Investigation of Crystallisation Kinetics in the LS-MEX Process

The differential temperature fields in the LS-MEX process have a significant influence on the crystallisation kinetics and, therefore, require a comprehensive investigation. This is based on a production example from Table 1, which only considers the standard production process without additional peripheral equipment. Specific positions within the extrusion strand, described in Section 3.1 and Section 3.3, are used to study the crystallisation process. Exothermic crystallisation effects are not considered in this study, particularly in the context of thermal process simulation.

The dynamics of the crystallisation kinetics are studied using temperature curves from thermal process simulations, supplemented by semi-empirical crystallisation models. Cooling and heating rates are determined by deriving these curves over time. The relative degree of crystallisation is based on the bimodal Nakamura model, whose cooling-rate-dependent parameters are determined by linear interpolation and extrapolation. The combination of these parameters with the simulated cooling rates allows the relative degree of crystallisation to be calculated using the differential form of the Nakamura model. The crystallisation rate results from the change in the degree of crystallisation with time, while the absolute degree of crystallisation is determined using the method described in Section 2.3.

**Figure 11:**
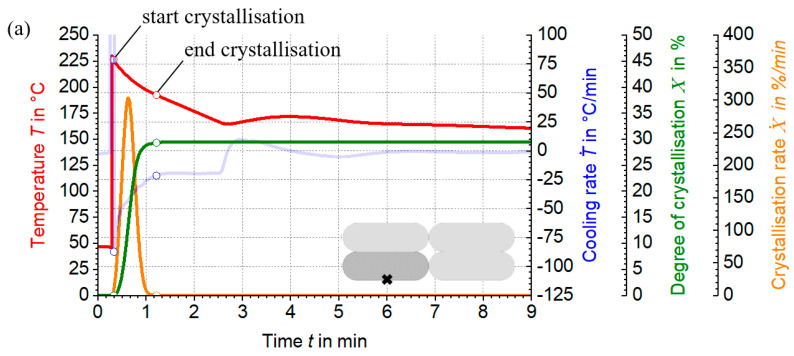

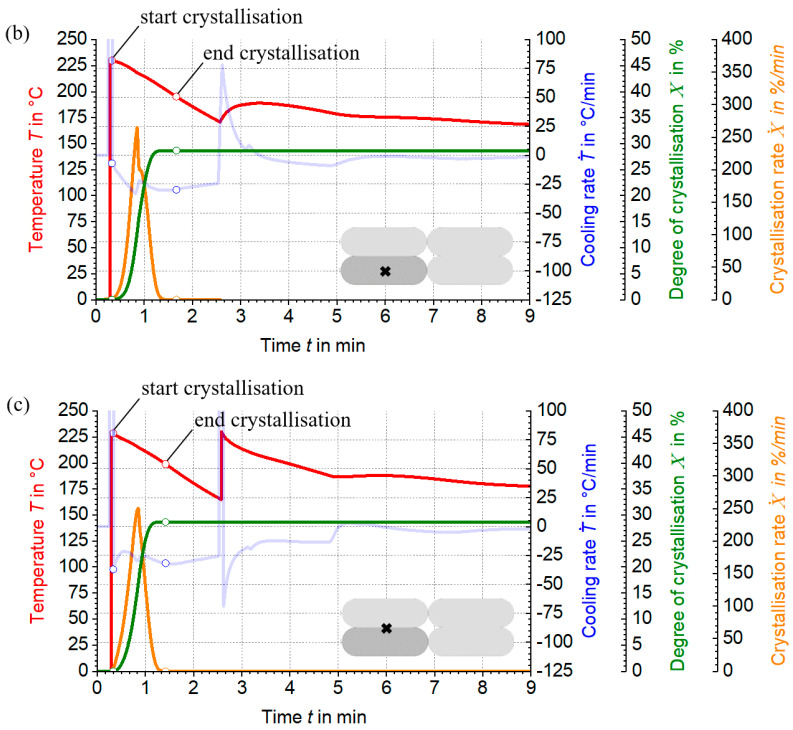
Progression of thermal profiles (red), cooling rate evolution (blue), crystallisation kinetics dynamics (green) and crystallisation rate evolution (orange) for different (**a**–**c**) material points in a strand.

Analysis of the temperature and cooling rate curves at different positions in the strand reveals differences over time, see Figure 11. Cooling is the slowest in the core of the extrusion strand (Figure 11b), which is reflected in lower cooling rates. In contrast, the material point at the print bed initially cools rapidly due to the temperature difference between the printbed and the strand. The cooling rate then decreases rapidly due to the low thermal conductivity of the print bed (Figure 11a). Cooling rates at the material point on the surface are increased compared to other positions (Figure 11c). Despite direct contact with the ambient temperature, the heat flow from the core results in a reduced cooling rate, highlighting the need to consider the thermal gradient in the LS-MEX process.

It can be seen that the absolute degree of crystallisation increases from the surface of the extrudate strand ($X_{Abs}$ = 28.56%) through the core ($X_{Abs}$ = 28.68%) to the point close to the print bed ($X_{Abs}$ = 29.32%), reflecting the specific boundary conditions of each material point. The differences in crystallinity are due to the thermal gradient in the extrudate strand. Observation of the crystallisation rate curves shows that the crystallisation rate increases as the cooling rate decreases. A sharp bend in the crystallisation rate peak in Figure 11b,c is caused by transient heating rates, which weaken the crystallisation process.

The analysis of the initial and final crystallisation values shows that the crystallisation process is completed more quickly in variant Figure 11a than in the other variants, due to the absence of additional heating rates which would otherwise delay the process. Nevertheless, under certain conditions, such as the choice of production parameters, the absolute degree of crystallisation is highest in Figure 11a.

In addition, the crystallisation process is completed before the next layer is deposited over the extrudate strand. This can restrict molecular diffusion of the overlying layer, particularly on the surfaces, and thus reduce adhesion between the strands [16]. The thermal energy introduced by the newly deposited extrudate strand could dissolve crystalline structures in the underlying layer or cause molecular chains to vibrate. These effects require further investigation to fully document the crystallisation history in the LS-MEX process.

## 4. Conclusions

This study underlines the crucial role of process parameters and peripheral equipment, heating and cooling elements in influencing the microstructural behaviour of materials, in particular, the crystallisation kinetics. The application of an additional cooling element significantly modifies the temperature profiles along the extrudate strand, as shown in Figure 10, and inhibits the formation of crystalline structures. This modification improves the diffusion of the molecule chains and the strand adhesion, thus improving the mechanical properties, a result confirmed by Tagscherer et al. [15].

The integration of simulation models with semi-empirical approaches allows a precise prediction of the microstructural dynamics (see Figure 11). This significantly reduces the dependence on extensive experimental testing. By using FEM simulations and adjusting production parameters, the study effectively evaluates crystallisation at different process stages. As described in Section 3.4, the crystallisation intensity increases from the strand surface to the print bed, which reflects the need to consider thermal gradients within the LS-MEX process that lead to different crystallisation degrees and structures.

However, these results are somewhat limited by the exclusion of the crystallisation kinetics during melting, indicating the need for improvements to the mathematical model [38]. Furthermore, the study emphasises the need for a more precise description of the temperature-dependent crystallisation constant within the Nakamura model, suggesting that the current assumption of constant values for the Avrami constant n and the temperature-dependent crystallisation constant $K\left(T\right)\;$is only a preliminary estimate.

Adjustments to production parameters, including the strategic use of peripheral equipment, allow controlled crystallisation to be used to set the desired mechanical properties. In particular, the improved adhesion between the extrudate strands leads to significantly better mechanical results of the final product, as shown in [16,37].

In summary, this study demonstrates that the targeted control of crystallisation kinetics by simulation and semi-empirical methods in combination with methodical parameters and equipment adjustments effectively optimises the properties of the end product. This approach not only increases the efficiency of production but also promotes the sustainability of the production process.

## 5. Outlook

The aim of this study is to provide a deeper insight into microstructural processes, in particular crystallisation dynamics, through the combined use of thermal simulations and semi-empirical approaches. It is shown that the integration of both methods allows a more accurate description and targeted modification of the crystallisation dynamics in LS-MEX to achieve the desired mechanical properties of the components.

To accurately reproduce the thermal behaviour in the manufacturing process, further validation studies between simulation and experimental results are required. These should provide reliable results over a wide range of process parameters and confirm the effect of additional peripheral equipment on the temperature field across the cross-section of the extrudate strand.

The semi-empirical Nakamura model for describing crystallisation kinetics requires a more detailed formulation to give more realistic results. While the Ziabicki approach is suitable for symmetrical peak profiles, the common asymmetrical peaks in semi-crystalline thermoplastics require the development of improved modelling approaches.

Experimental validation studies are essential to confirm the crystallisation process in LS-MEX and support the combined use of thermal simulation and semi-empirical approaches to describe crystallisation.

Understanding the history of the crystallisation process in LS-MEX is essential to elucidate the relationship between the mechanical properties of the final product and the resulting microstructural processes. Mechanical tests should be carried out to identify and verify these relationships, thereby providing a more complete understanding of the material behaviour during the manufacturing process.

## Figures and Tables

**Table 1:** Summary of manufacturing parameters, dimensions and values for thermal boundary conditions in the simulation example.

Manufacturing Parameters	Unit	Value
Extrudate temperature, $T_{Ext}$	°C	230
Print bed temperature, $T_{Bed}$	°C	47
Ambient temperature, $T_{Amb}$	°C	26
Printing speed, v	m/s	$1.5\cdot 10^{-2}$
Layer width, $L_{W}$	m	$13.5\cdot 10^{-3}$
Layer height, $L_{H}$	m	$4.5\cdot 10^{-3}$
Power density—Cooling element, ${\vec{q}}_{CP}$	$W/{(m}^{2}K)$	$-13\cdot 10^{4}$
Power density—Heating element, ${\vec{q}}_{HP}$	$W/{(m}^{2}K)$	$13\cdot 10^{4}$
Nozzle-periphery distance, $s_{NP}$	m	$20.25\cdot 10^{-3}$
**Manufactured Part Dimensions**	**Unit**	**Value**
Length, L	m	$5\cdot 10^{-1}$
Width, W	m	$5.4\cdot 10^{-2}$
Height, H	m	$2.25\cdot 10^{-2}$
**Thermal Boundary Condition**	**Unit**	**Value**
Heat transfer coefficient—Polymer, $h_{convPo}$	$W/{(m}^{2}K)$	13.45
Heat transfer coefficient—Print bed, $h_{convPb}$	$W/{(m}^{2}K)$	5
Emissivity, ε	−	1
**Discretisation Parameters**	**Unit**	**Value**
Mesh length and width, $M_{L}$ and $M_{W}$	m	$6.75\cdot 10^{-3}$
Mesh height, $M_{H}$	m	$2.25\cdot 10^{-3}$
Time step, ∆t	s	0.5

## Data Availability

Data are contained within the article.

## Abbreviations

Large Scale Material Extrusion	LS-MEX
Polyamide 6 with 40% carbon fibres	PA6 wt.%40 CF
Finite element method	FEM

## Appendix A. Material Properties for Thermal Process Simulation

**Table A1:** Thermophysical characterisation of the print bed material: wood according to ANSYS—Tabular representation of the thermal properties.

Thermal Properties Wood	Unit	Value
$\mathrm{Specific}\;\mathrm{heat}\;\mathrm{capacity},\;c_{p}$	J/(kgK)	1800
Thermal conductivity, λ	W/(mK)	0.18
